# Book Reviews

**Published:** 1874-09-15

**Authors:** 


INFLAMMATION OF THE LUNGS, TUBERCULOSIS AND CONSUMPTION. Twelve Lectures by Ludwig Buhl, Professor of Pathological Anatomy, etc., in the University of Munich. Translated by Mathew D. Mann, M.D., and S. B. St. John, M.D. New York: G. P. Putnam & Sons.

In the twelve lectures here grouped together, we have a clear, concise exposition of the author's views regarding the pathology, histology, and morbid anatomy of the various pulmonary diseases. His views regarding the origin of phthisis and tuberculosis differ materially from those set forth by Niemeyer and his followers, and which have been so generally accepted by the profession.

These theories evolved by Prof. Buhl, as the result of long and careful investigation and observation, have won to their support many of the most prominent German pathologists, notably among others, Prof.

Rindfleisch, whose excellent work on pathological anatomy is familiar to American students.

The lectures constitute a 12-mo. volume of about 160 pages.

---

ARCHIVES OF OPHTHALMOLOGY AND OTOLOGY. Edited and published in English and German by Prof. H. Knapp, M.D., New York, and Prof. S. Maas, M.D., Heidelberg, assisted by Dr. E. Gruenny, New York, and Dr. C. J. Blake, Boston. New York: Wm. Wood & Co.

The first number of the fourth volume of this journal is received. It is hereafter to be issued quarterly instead of semi-annually—the numbers to contain from 112 to 160 pages each. About four-fifths of the space will be devoted to original communications, lectures, etc., and the remainder to reviews. The present number contains a large amount of valuable and interesting matter pertaining to this special department, and is handsomely illustrated by lithograph plates.

---

NOMENCLATURES OF DISEASES. Prepared for the use of the Medical Officers of the United States Marine Hospital Service. By the Supervising Surgeon, J. M. Woodworth, M.D.

This is a simple republication, in convenient form for the use of the marine hospital service, of the Provisional Nomenclature of Disease, as drawn up by a joint committee appointed by the Royal College of Physicians of London, Eng.

This classification has received the endorsement of the American Medical Association, and of the American Public Health Association.

---

ESSAYS ON CONSERVATIVE MEDICINE AND KINDRED TOPICS. By Austin Flint, M.D. Philadelphia: H. C. Lea.

This is a small volume of 214 pages, made up of a series of essays which have appeared at various times in the medical journals. As here gathered together they form an interesting little volume, which will be read with profit by many to whom they have not previously been accessible.

---

THE PSYCHOLOGICAL AND MEDICO-LEGAL JOURNAL. Conducted by Wm. A. Hammond, M.D., and T. M. B. Cross, M.D. New York: F. W. Christian, 77 University Place.

The first number of the new issue of this special journal is also before us. It is to be issued in monthly numbers, commencing with July, 1874. Subscription price, $5.00 per annum.

